# Concomitant Pathogenic Mutations and Fusions of Driver Oncogenes in Tumors

**DOI:** 10.3389/fonc.2020.544579

**Published:** 2021-01-15

**Authors:** Runjiao Zhang, Li Dong, Jinpu Yu

**Affiliations:** ^1^Cancer Molecular Diagnostics Core, Tianjin Medical University Cancer Institute and Hospital, National Clinical Research Center of Caner, Key Laboratory of Cancer Prevention and Therapy, Key Laboratory of Cancer Immunology and Biotherapy, Tianjin, China; ^2^Tianjin's Clinical Research Center for Cancer, Tianjin, China

**Keywords:** concomitant, mutation, fusion, lung cancer, thyroid cancer, leukemia

## Abstract

Driver oncogene alterations have always been one of leading causes in the process of occurrence and development of tumors. And the effects of driver oncogene alterations on tumorigenesis and progression in different kinds of tumors have been studied heatedly. And the roles that the driver oncogenes alterations play have been elucidated clearly in previous studies. The phenomenon of concomitant driver oncogenes mutations and driver genes fusions has gained much concentration in the past two decades. And a growing number of studies reported this phenomenon, either coexistence or mutually exclusivity. Here we reviewed on the phenomenon of concomitant mutations in three common types of carcinomas—lung cancer, thyroid cancer, and leukemia, which have been studied relatively more detailed and more general compared with others.

## Introduction

Genetic mutations are an important molecular background in tumors, and the most common types of alterations are point mutations and fusions. Currently, a large number of researches have been conducted to deeply study the influences of driver oncogenes (e.g. *ALK*, *RAS*, *RAF*, etc.) on the occurrence and development of different kinds of cancers. It has been correspondingly clear that driver oncogene mutations play diverse roles and are of vital importance in different types of tumors in the process of carcinogenesis, development, invasiveness, and metastasis. Also, effects of common driver gene fusions (e.g. *RET* fusion, etc.) on the oncogenesis and progression of different kinds of tumors have been demonstrated clearly by lots of studies. Nevertheless, studies and cognitions on the phenomenon of concomitant pathogenic mutations and fusions of driver oncogenes in tumors are relatively limited and not very completely investigated. Furthermore, in different types of carcinomas, dual driver mutations and driver gene fusions has different effect on the occurrence, development, invasiveness, and metastasis of tumors; what’s more, the occurrent frequency of this dual mutations and fusions in diverse neoplasms is also different. A portion of studies have been conducted to study simultaneous proto-oncogene mutations and driver gene fusions in different kinds of tumors in the past few decades. Herein we review on the coexistence of pathogenic mutations and fusions of driver oncogenes in lung cancer, thyroid cancer and leukemia, in which this phenomenon have been identified. Furthermore, we discuss the influences on tumors of this phenomenon, including clinical pathological features and prognosis of patients harboring dual mutations and fusions.

## Concomitant Oncogene Mutations and Rearrangements in Lung Cancer

Lung cancer is the most commonly diagnosed carcinoma among different kinds of malignancies and is also the leading cause of cancer-related death both in China and worldwide (1–4). Lung cancer consists of two groups, small cell lung cancer (SCLC) and non-small cell lung cancer (NSCLC). And NSCLC is further divided into subtypes including large cell carcinoma (LCC), squamous cell lung cancer (SC), and adenocarcinoma (AC) (5). A large proportion of lung cancers are associated with tobacco smoking, while evidence shows that lung cancer in lifelong non-smokers appears to be a distinct disease caused by oncogene driver mutations which are different from the genetic pathways observed in lung cancer of smokers (6–13).

Epidermal growth factor receptor (*EGFR*) is a transmembrane protein with cytoplasmic kinase activity that transduces important growth factor signaling from the extracellular milieu to cells (14). And *EGFR* mutations are common in lung cancer. Driver genomic fusions [e.g. anaplastic lymphoma kinase (*ALK*), *ROS1*, and *KIF5B-RET* fusion, etc.] are thought not to be correlated with smoking-associated mutations and frequently served as driver events of smoking-signature-low lung adenocarcinomas. *EGFR* mutations are more common in Asian women, while *ALK* fusions showed no gender or racial difference which the age of onset was younger, and occurred more commonly in mucus-type lung cancer in pathology. The phenomenon of coexisting driver mutations and fusions has conventionally been considered to be mutually exclusive (15–25). However, cumulative studies have revealed that concomitant occurrence of driver gene mutations and gene fusions accounts for a small number of NSCLC cases (26–37). And concomitant *EGFR* mutations and *ALK* fusions is the most common form among all kinds of coexistence of driver mutations and driver oncogene fusions in NSCLC. In accordance with ALK fusions in lung cancer, the phenomenon of dual driver mutations and gene fusions has also been detected and elucidated in the never smoking patients with NSCLC (mainly lung adenocarcinomas) in several studies. Zhao et al. recruited 5,816 patients with lung cancer from Shanghai, China, and all of the patients are asked to undergo both *EGFR* mutation and ALK fusion analysis. They found that 2,392 (41.1%) patients had *EGFR* mutations, 503 (8.7%) had *ALK* fusions, and 26 (0.45%) had simultaneous *EGFR* mutations and *ALK* fusion (38). Won et al. analyzed the *EGFR* and *ALK* status in 1,458 cases of lung cancer including NSCLC (n = 1,445) and small-cell carcinoma (n = 13) using direct sequencing and FISH, respectively, and the cohort enrolled in the study are from Seoul, Korea. In this cohort, the *EGFR* mutation and *ALK* fusion rates in NSCLC patients were 42.4% (612/1,445) and 6.3% (91/1,445), respectively, and concomitant *EGFR* and *ALK* alteration was detected in 4 (0.3%) of the 1,445 NSCLC patients (35). Yang et al. carried out a study in Guangdong, China, which screened a total of 977 patients with NSCLC for the presence of *EGFR* mutations, *ALK* fusion and coexistence of *EGFR* mutations and *ALK* fusion. And 336 (34.4%) and 70 (7.2%) patients had *EGFR* mutations or *ALK* fusion, respectively. Meanwhile, thirteen (1.3%) patients harbored concomitant *EGFR* mutations and *ALK* fusion, all of which were adenocarcinomas and never or light smokers (36). Lee et al. detected *EGFR* mutations and *ALK* fusion among 444 Korean lung adenocarcinoma patients. They found 228 (51.4%) patients harbored EGFR mutations and 34 (7.7%) had *ALK* fusion, meanwhile four patients (0.9%) were found to have both *EGFR* mutations and *ALK* fusion (**Table 1**) (6).

**Table 1:** Summary of studies on concomitant oncogene mutations and rearrangements in lung cancer.

Study		Samples	Molecular biomarkers			Methodology
			EGFR mutations	ALK fusions	EGFR mutations & ALK fusions	
1	Lou et al.	118	84 (71.2%)	23 (19.5%)	11 (9.3%)	Mutation:direct sequencing and amplification refractory mutation system (ARMS) Fusion:FISH and/or IHC
2	Zhao et al.	5,816	2,392 (41.1%)	503 (8.6%)	26 (0.5%)	
3	Won et al.	1,445	612 (42.4%)	91 (6.3%)	4 (0.3%)	
4	Lee et al.	444	228 (51.4%)	34 (7.7%)	4 (0.9%)	
5	Fan et al.	synchronous multifocal LUAC:97; unifocal LUAC:962	61/97 (62.89%) 570/962 (59.25%)	14/97 (14.43%) 62/962 (6.44%)	4/97 (4.71%) 8/962 (0.83%)	
6	Yang et al.	977	336 (34.4%)	70 (7.2%)	13 (1.3%)	Mutation:direct sequencing Fusion:RT-PCR and RACE-PCR

Lung cancer.

With the widespread popularization and utilization of low-dose chest computed tomography (CT) as early-stage lung cancer screening, the incidence reported of lung cancer patients who present with multiple lesions ranges from 0.2 to 20%, especially those of multiple lung adenocarcinomas which is a rare molecular subtype of lung adenocarcinomas. A research launched by Fan et al. collected 1,059 patients with lung adenocarcinomas from Hubei, China to detect *EGFR* and *ALK* alterations. A total of 97 multiple synchronous lesions were observed among 1,059 LUAC patients, among which patients with concomitant *EGFR* mutations and *ALK* fusion were 4.12% (4/97). Comparatively, patients with unifocal LUAC harboring *EGFR/ALK* co-alterations were 0.83% (8/962). Apparently, the incidence of *EGFR/ALK* co-alterations in the multifocal LUAC was significantly higher than that in the unifocal LUAC (39). In accordance with the study by Fan et al., another study from Shanghai, China by Wu et al. reported that the rate of *EGFR/ALK* co-alterations in patients with synchronous multiple lung ground-glass opacity nodules was as high as 8.57% (40).

Although concomitant *EGFR* mutations and *ALK* fusion in lung cancer had once been considered to be mutually exclusive, the coexistence of *EGFR* mutations and *ALK* fusion in patients with lung cancer has been detected in a series of studies in recent years. Most of these studies are clinical researches, and their research contents mainly include the three aspects below: the correlation between concomitant mutations and clinical pathologic features of patients, influence of dual mutations on patients’ prognoses, potential mechanisms of TKI resistance and guidance of therapeutic regimens.

In regard to the relationship between the status of *EGFR* and *ALK* (*EGFR* mutations, *ALK* fusions and *EGFR/ALK* co-alterations) and the clinical pathological features of patients with lung cancer, there are not many studies conducted. Lou et al. reported 11 patients with dual mutations, 84 *EGFR* mutations and 23 *ALK*-positive patients with median survival time of 18.5, 21.3, and 23.7 months (p = 0.06), respectively. There was no statistical difference among the three groups, but the survival time of patients with dual mutations was the shortest, and there was a significant difference between *ALK*-positive group and *EGFR/ALK* co-alterations group (29). Elisa Brega and Guilherme Brandao also support the results that patients with more than two gene alterations living shorter than those with no or one gene alteration. Although the overall survival (OS) of patients with coexistent mutations is shorter than that of patients with single alteration of *EGFR* or *ALK*, the prognosis of patients with dual mutations is still better than that of the patients receiving chemotherapy alone after rational targeted therapy (41).

Main research content of the studies mentioned above is the responses to *EGFR* TKIs or/and *ALK* inhibitors among patients with concomitant *EGFR/ALK* alterations. First generation *EGFR* TKIs such as gefitinib, erlotinib, and icotinib provide survival benefits over conventional chemotherapy and have revolutionized the therapy of patients with NSCLC with *EGFR*-activating mutations, as has crizotinib, a TKI targeting *ALK* fusions, for *ALK*-positive patients. As for the efficacy of *EGFR* TKIs and crizotinib in *EGFR/ALK* double-positive patients, it has been controversial. Some reports have indicated that *EGFR* TKIs had a better response than *ALK* inhibitors in terms of objective response rate (ORR) and progression free survival (PFS) (27, 29, 30), but others have come to the opposite conclusion (31, 34, 35, 42). Wu et al. came to the conclusion that for NSCLC patients with coexistence of *EGFR* mutations and *ALK* fusion, first-line *EGFR* TKIs may be a reasonable care. Whether to administer application of crizotinib subsequently depends on *ALK* fusion status, and relative levels of *EGFR* and *ALK* phosphorylation (29). This may provide a new thinking that detecting the abundance of *EGFR* mutations and *ALK* fusion and the levels of phosphorylation of downstream proteins can determine whether or not to use sequential therapy or in combination with crizotinib, so that the most effective therapeutic regimens might be optimized for patients with concomitant *EGFR* and *ALK* alterations in the future. While their study is single-institution, small-sample, and retrospective, so their result can not accurately reflect the population with *EGFR/ALK* co-alterations at large. Zhao et al. found that first generation *EGFR* TKIs and *ALK* inhibitors in patients with concomitant *EGFR* mutations and *ALK* fusion were equally efficacious as in patients with single gene alterations. *EGFR/ALK* co-altered patients also appeared to have longer overall survival (OS) than patients with *EGFR* mutant disease after the sequential treatment with *EGFR* and *ALK* TKIs. So multi-drug combined therapy might be the best option for patients with simultaneous mutations. Nevertheless, the results have no statistical significance and this might be due to a small number of samples recruited were qualified to be included into the study (38).

The mechanisms of resistance to *EGFR* or *ALK* TKIs is another focus that researchers mainly concentrated on. Lou et al. demonstrated that relatively higher level of phospho-*EGFR* was the mechanism of resistance to crizotinib and the relatively higher level of phospho-*ALK* may be molecular mechanisms of resistance to *EGFR* TKIs (29). And Takaaki et al. held the same opinion that activation of *EGFR* signaling as a bypass signaling mechanism can contribute to *ALK* inhibitor resistance. In the presence of *EGF*, crizotinib was still able to inhibit *ALK* phosphorylation but not AKT, S6 and ERK1/2 phosphorylation (43). This can reasonably explain why single TKI (*EGFR* TKIs or *ALK* inhibitors) is not effective in the therapy of patients with dual mutations, and provide treatment advices for patients with concomitant mutations.

## Concomitant Oncogene Mutations and Rearrangements in Thyroid Cancer

Thyroid carcinoma is the most common type of endocrine malignancies and can be classified into papillary thyroid carcinomas (PTCs), follicular thyroid carcinomas (FTCs), undifferentiated carcinomas, medullary thyroid carcinomas (MTCs), Hürthle cell carcinomas (HTCs), and poorly differentiated or anaplastic thyroid carcinomas (ATCs) (44). And in all of the multiple types of differentiated thyroid carcinoma, PTC is the most common one which accounts for more than 80% of thyroid malignancies (45). Identified oncogene mutations in thyroid neoplasms mainly consist of *BRAF* and *RAS*. *BRAF* mutations are the most common genetic alteration occurring in thyroid carcinomas especially in PTC, accounting for 28–83%, with an overall rate of 44% (46–48). The most common type of *BRAF* mutations found in PTC is a T to A substitution at nucleotide 1,799 in exon 15, which results in the conversion of a valine to glutamic acid at codon 600 (V600E) of the *BRAF* protein (49, 50). All of the relevant studies mainly demonstrate the influence of *BRAF* V600E on thyroid cancer. *BRAF* mutation has been illustrated to be associated with poor prognosis among PTC patients (51–53). *BRAF* V600E mutation occurs more frequently in the advanced stages (stage III or IV) of thyroid cancer (54). And the association between advanced stages and *BRAF* V600E becomes significant while compared with tumors with all other oncogene mutations. Taking only BRAF mutation positive tumors into account, the percentage of *BRAF* V600E alleles was directly correlated with disease stages (55). Among patients with *BRAF* V600E, the age of preliminary diagnosis is relatively younger (<45 years old) than others (56).

Gene fusions detected in patients with thyroid cancer are mainly *RET* and *NTRK* fusions. And RET/PTC is one of the most common kind of fusions, which mainly consists of RET/PTC-1 (*CCDC6-RET*), RET/PTC-2 (*PRKAR1A-RET*), and RET/PTC-3 (*NCOA4-RET*) (57). RET/PTC1 and RET/PTC3 are intrachromosomal fusions of the long arm of chromosome 10 and can be induced *in vitro* by irradiating normal thyroid cells (58, 59). The incidence of RET/PTC fusions in patients with PTC ranges from 2.5 to 67.0% depending on the cohorts studied. Most patients with RET/PTC fusions in their primary PTCs were younger than 45 years of age (60–64). And the distribution of stages varies in different cohorts that had been studied.

The phenomenon of concomitant *BRAF* mutations and gene fusions in thyroid malignancies has been controversial up to now. Some scholars hold the opinion that dual *BRAF* mutations and gene fusions can exist in the same thyroid tumor. In addition, they deem that this phenomenon is associated with clinical pathological features of the tumor and it may become a prognostic indicator for patients with thyroid cancer. A study from Texas M. D. Anderson Cancer Center by Henderson et al. reported concomitant *BRAF* and RET/PTC mutations in 5/54 (9.3%) recurrent PTC. And the prevalence of tumors with this concomitant driver mutations and fusions found in the recurrent population far exceeded the frequency historically reported for patients with primary PTC. They also found that patients with dual mutations and fusions were significantly older and had more advanced tumors than patients with a *BRAF* mutation or RET/PTC alone (56). Guerra et al. recruited 72 patients from Salerno, Italy and they demonstrated that patients with both *BRAF* mutations and RET/PTC accounted for 19.4% (14/72) among all of the patients of PTC. Different from Henderson’s discovery, they found that tumors with dual mutations were equally distributed among stages. And they found that the presence of dual mutations is associated with a higher rate of recurrence (55). Zou et al. found six patients with CPTC and one with tall-cell variant to have simultaneous *BRAF* V600E and RET/PTC-1 from an 85-participant cohort. What they found is consistent with the discovery of Henderson, all the seven patients with concomitant *BRAF* V600E and RET/PTC-1 were in the advanced stages (stage III or IV). And the association of concomitant mutations and fusions with advanced stages was statistically significant. They also elucidated that patients with concomitant mutations and fusions had much poorer clinical outcomes than those with a single or no mutation (**Table 2**) (54).

**Table 2:** Summary of studies on concomitant oncogene mutations and rearrangements in thyroid cancer.

Study		Samples	Molecular biomarkers			Methodology
			BRAF V600E	RET/PTC	BRAF V600E&RET/PTC	
1	Henderson et al.	54	42 (77.8%)	9 (16.7%)	5 (9.3%)	Mutation:Pyrosequencing
2	Guerra et al.	72	32 (44.4%)	26 (36.1%)	14 (19.4%)	
3	Zou et al.	85	42 (49.4%)	9 (11.0%)	7 (8.2%)	
4	Bastos et al.	118	42 (35.6%)	29 (27.1%)	3 (2.5%)	Fusion:RT-PCR

Thyroid cancer.

Nevertheless, others think that gene fusions are mutually exclusive with *BRAF* mutations in patients harboring PTC, as well as with each other. And mutations at more than one of these genes are unlikely to provide an additional biological advantage. Paula et al. conducted a study including 50 patients with PTC from Porto, Portugal and detected 46% (23/50) of them harboring *BRAF* V600E. And meanwhile, seven of 39 PTC (18%) were validated to have RET/PTC fusions; none of these 7 PTC cases had the *BRAF* V600E mutation. So they thought that *BRAF* V600E mutation appeared to be an alternative event to RET/PTC in PTC (65). Liang et al. detected the mutation conditions of *BRAF* mutations and *RET* fusions among 355 Chinese patients with primary PTC. They found 72.4% (257/355) of the cases carried *BRAF* mutations and 8.5% (30/355) of cases were characterized with in-frame gene fusions. In order to further validate whether BRAF mutations is mutually exclusive with *RET* fusions, they screened patients with known *BRAF* point mutations for *RET* fusions transcripts by using RT-PCR, and none of *RET* fusions was identified in patients with *BRAF* point mutations (66). The Cancer Genome Atlas Research Network analyzed 496 patients with thyroid malignancies [324 (65.3%) classical-type, 99 (20.0%) follicular-variant, 35 (7.1%) tall-cell variant, 9 (1.8%) uncommon PTC variants and 29 without histological annotations] in The Cancer Genome Atlas (TCGA) project by comprehensive multi-platform analysis and drew the conclusion that fusions were mutually exclusive with each other and with proto-oncogenes mutations (67).

## Concomitant Oncogene Mutations and Rearrangements in Leukemia

Leukemia is a genetically and clinically heterogeneous disease (68, 69). It consists of four main subtypes: acute myeloid leukemia (AML), acute lymphocytic leukemia (ALL), chronic myeloid leukemia (CML), and chronic lymphocytic leukemia (CLL). Genomic aberrations are known to play an important role in the pathogenesis of leukemia, and cytogenetic aberrations have become well established diagnostic and prognostic markers. In the past few decades, mounting gene alterations have been detected and proved to play a role in the occurrence and development of leukemia. Meanwhile, some co-occurring mutations and fusions have also been found, especially in acute leukemia (AML and ALL). Hidemasa et al. recruited pediatric patients with MLL-rearranged (MLL-r) AML (n = 56) alongside data from the TARGET study’s pediatric cohorts with MLL-r AML (n = 104), non–MLL-r AML (n = 581), and adult MLL-r AML (n = 81) into their study, and they found that KRAS mutations were most frequent in pediatric patients with high-risk MLL fusions (MLL-MLLLT10, MLL-MLLT4, and MLL-MLLT1), accounting for 26.25% (42/160) in MLL-r AML. What’s more, an adverse prognostic impact of KRAS mutations was confirmed in adult MLL-r AML; KRAS mutations were associated with adverse prognoses in pediatric patients with both high-risk (MLLT10+MLLT4+MLLT1; n = 60) and intermediate-to-low-risk (MLLT3+ELL+others; n = 100) MLL fusions (70). The t (8;21) translocation is one of the most frequent cytogenetic abnormalities in acute myeloid leukemia (AML), resulting in the RUNX1/RUNX1T1 fusion. The transcription factor ZBTB7A is important for hematopoietic lineage fate decisions and for regulation of glycolysis. On a functional level, ZBTB7A mutations disrupt the transcriptional repressor potential and the anti-proliferative effect of ZBTB7A. Luise et al. revealed a kind of concomitant mutations and fusions - ZBTB7A mutations with t (8;21) translocation. ZBTB7A acts as a tumor suppressor in RUNX1-RUNX1T1 AML, so the specific association of ZBTB7A’s loss-of-function-mutations with t (8;21) rearranged AML can point towards leukemogenic cooperativity between mutant ZBTB7A and the RUNX1/RUNX1T1 fusion (71). And in their cohort, 13 (23.2%) samples were found to have ZBTB7A mutations among 56 AML patients with RUNX1-RUNX1T1. In addition, concomitant ZBTB7A mutations with t (8;21) translocation has also been reported in many relevant articles accounting for 4.8–23.2% in patients with RUNX1-RUNX1T1 fusion (**Table 3**) (72–76).

**Table 3:** Summary of studies on concomitant oncogene mutations and rearrangements in leukemia.

Study		Samples	Molecular biomarkers			Methodology
			ZBTB7A mutations	RUNX1/RUNX1T1	ZBTB7A mutations & RUNX1/RUNX1T1	
1	Luise et al.	56	13 (23.2%)	56 (100%)	13 (23.2%)	Mutation:DNA sequencing Fusion:RNA sequencing
2	Zachary et al.	165	8 (4.8%)	85 (51.5%)	8 (4.8%)	whole-genome sequencing or whole-exome sequencing
3	Naomi et al.	41	4 (9.8%)	41 (100%)	4 (9.8%)	Mutation:DNA sequencing Fusion:RQ-PCR
4	Friederike et al.	331	43 (13%)	331 (100%)	43 (13%)	Mutation:DNA sequencing Fusion:FISH
5	Sabrina et al.	292	31 (10.6%)	130 (44.5%)	28 (9.6%)	Whole-exome sequencing or custom-targeted sequencing

Leukemia.

## Concomitant Oncogene Mutations and Rearrangement in Other Solid Malignancies

The phenomenon of concomitant mutations and fusions of driver genes is relatively uncommon compared with single driver oncogene mutations and fusions. However, with the deepening of the sequencing depth in next generation sequencing technology and a wider application range of NGS, more and more dual mutations and fusions have been noticed by scholars. The most obvious is in lung cancer, thyroid cancer and leukemia, a fair amount of studies focused on this phenomenon and conducted corresponding clinical studies. Although there are relatively few articles reporting the co-occurrence of driver mutations and fusions in other solid tumors, like breast cancer, colorectal cancer, prostate cancer and melanoma, some cases of concomitant mutations and fusions of driver genes have attracted scholars’ attention.

Mutations in BRCA1 confer a high risk for breast cancer, and BRCA1 is involved in many cellular processes as well as in repairing double-stranded DNA breaks (DSBs) mediated by homologous recombination (77–79). Kevin et al. and Stephens et al. deemed that deficiencies in BRCA1 would cause increased chromosomal instability in a tumor cell due to impaired DNA repair pathways and NHEJ dysfunction. The resulting chromosomal lesions may potentially lead to the creation of gene fusions that can be detected in the transcriptome. And they found the concomitant BRCA1 mutations and gene fusions in breast cancer cells (80, 81). Regretfully, no corresponding large sample clinical study was conducted to identify this phenomenon. Similarly, Manuel et al. held the opinion that defects in DNA repair may lead to an increase of chromosomal rearrangements and thus to the occurrence of the somatic fusion of TMPRSS2 to ETS oncogenes in prostate cancer, and they detected that DNA repair genes (BRCA2, ESCO1, and POLI, etc.) mutations exist in TMPRSS2-ERG fusion-positive samples (82). In addition, no pathogenic mutations and fusions of driver genes has been found in breast cancer and prostate cancer up to now. Activation of RAS/MAPK pathways through mutations of RAS family members and BRAF are classical driver mutations of colorectal cancer (83). And NTRK1 and NTRK3 fusions have been described as oncogenic driver alterations in colorectal cancer (84–87). While the studies on the genomic landscape of colorectal cancers revealed that NTRK fusions are mutually exclusive with driver mutations like KRAS and BRAF mutations (85, 88–90). In melanoma, James et al., Iwei et al., and Thomas et al. thought that fusion proteins and most common oncogenic drivers such as BRAF, NRAS and HRAS are mutually exclusive, so that NTRK fusion proteins might be more common in BRAF or NRAS wild-type melanoma (91–93). However, there is still one study conducted by Lezcano et al. detected a case that harbored NTRK1 fusion as well as an additional activating NRAS Q61 mutation (94).

## Disparity in the Incidence Rates of Concomitant Driver Mutations and Fusions

The phenomenon of concomitant mutations and fusions in cancer indeed exists. The incidence rates of concomitant driver mutations and fusion vary in cancers, while the frequency is relatively low, and associated with different type of cancers or gene varieties. The possible reasons affecting the incidence of concomitant mutations and fusions are as follows: (1) Ethnic factors: Concomitant driver mutations and fusions may have racial differences. And the racial differences mainly reflect in NSCLC. Among all the studies about NSCLC mentioned above, four of the five cohorts are from China and one from Korea, that is all the data are from Asians, which may suggest that concomitant driver mutations and fusions in NSCLC is more common in Asian populations. (2) Detecting technologies: The methods and platforms that are utilized in the detection of fusions varied in different studies, and these methods’ interval of sensitivity has a wide range. The incidence of dual driver mutations and fusions may be low by direct sequencing and FISH, while next generation sequencing can increase the detection rate. And the reaction conditions and primer selection in the detection of fusions are main influence factors, and inappropriate conditions and primer may lead to false positive or false negative results, thus bringing out incorrect judgements. It is believed that with the improvement of detection depth and sensitivity, the incidence of concomitant driver mutations and fusions will increase in the future. (3) Sample selection: DNA samples are usually applied to mutation detection while fusion detection requires RNA samples. Most of the samples extracted from tumors are single sample type—DNA or RNA, so that mutations and fusions cannot be detected appropriately at the same time. If DNA and RNA could be detected simultaneously, more samples with concomitant driver mutations and fusions will be found. (4) Population inclusion: Patients with different subtypes of tumors and different stages may present disparate genotypes. In NSCLC patients, most of the dual mutations have been reported in advanced and/or metastatic patients in the literatures, and this may be the result of the accumulation of genetic changes during the development of the disease. Therefore, the selection of different stages of samples may lead to totally opposite conclusions. So, these questions need to be taken into account in the following researches. (5) Source of tissue: We found that the phenomenon of concomitant mutations and fusions of driver genes are mainly detected in lung cancer, thyroid cancer, and leukemia. These three types of carcinomas are more susceptible to carcinogenic factors. Lung cancers are vulnerable to tobacco-smoking, thyroid cancers are closely related to radiation, and leukemia may be associated with chemicals. And in the process of exposure to different carcinogenic factors, there tends to be genomic instability in cancer cells, leading to an increased frequency of gene fusions. Conversely, for other carcinomas caused by intrinsic factors, gene fusions may be relatively uncommon.

## Clinical Significance of Concomitant Driver Mutations and Fusions

In summary, the concomitant driver mutations and fusions may occur in specific molecular subtypes of tumor and advanced-stage tumors, and it is recommended to carry out a multiple centers-based large sample retrospective study by multi-point biopsy or liquid biopsy combined with next generation sequencing (NGS), so as to obtain the precise results of the frequency of concomitant mutations and fusions, and further to identify the correlation with multiple clinical and prognostic features. Besides, functional experiments and animal models are necessary to explore whether there are differences in biological behaviors and signaling pathway activated between concomitant driver mutations and fusions and single driver gene alterations. Therefore, we can lay the theoretical foundation for the occurrence of this phenomenon. In this way, the clinical significance of concomitant mutations and fusions should be elucidated and individualized target therapy should be proposed to benefit more patients from the development of precision medicine.

## Author Contributions

RZ and JY conceptualized and designed the study. RZ wrote the article. LD critically revised the article. JY gave the final approval of the article. All authors contributed to the article and approved the submitted version.

## Funding

This work was supported by National Natural Science Foundation of China (Grant No. 81702280, 81472473, 81872143), National Science and Technology support Program of China (Grant No. 2015BAI12B15, 2018ZX09201015), National Key Research and Development program of China: The Net construction of human genetic resource Bio-bank in North China (2016YFC1201703), Projects of Science and Technology of Tianjin (Grant No. 13ZCZCSY20300, 18JCQNJC82700) and Key project of Tianjin Health and Family Planning Commission (Grant No. 16KG126).

## Conflict of Interest

The authors declare that the research was conducted in the absence of any commercial or financial relationships that could be construed as a potential conflict of interest.
